# Crystal structures of the S6K1 kinase domain in complexes with inhibitors

**DOI:** 10.1007/s10969-014-9188-8

**Published:** 2014-07-31

**Authors:** Hideaki Niwa, Junko Mikuni, Shunta Sasaki, Yuri Tomabechi, Keiko Honda, Mariko Ikeda, Noboru Ohsawa, Motoaki Wakiyama, Noriko Handa, Mikako Shirouzu, Teruki Honma, Akiko Tanaka, Shigeyuki Yokoyama

**Affiliations:** 1 RIKEN Systems and Structural Biology Center, 1-7-22 Suehiro-cho, Tsurumi, Yokohama, 230-0045 Japan; 2 RIKEN Center for Life Science Technologies, 1-7-22 Suehiro-cho, Tsurumi, Yokohama, 230-0045 Japan; 3 Teijin Institute for Bio-Medical Research, Teijin Pharma Ltd, 4-3-2, Asahigaoka, Hino-shi, Tokyo 191-8512 Japan; 4 RIKEN Structural Biology Laboratory, 1-7-22 Suehiro-cho, Tsurumi, Yokohama, 230-0045 Japan

**Keywords:** Protein kinases, Inhibitors, p70 S6 kinase, S6K1

## Abstract

Ribosomal protein S6 kinase 1 (S6K1) is a serine/threonine protein kinase that plays an important role in the PIK3/mTOR signaling pathway, and is implicated in diseases including diabetes, obesity, and cancer. The crystal structures of the S6K1 kinase domain in complexes with staurosporine and the S6K1-specific inhibitor PF-4708671 have been reported. In the present study, five compounds (F108, F109, F176, F177, and F179) were newly identified by in silico screening of a chemical library and kinase assay. The crystal structures of the five inhibitors in complexes with the S6K1 kinase domain were determined at resolutions between 1.85 and 2.10 Å. All of the inhibitors bound to the ATP binding site, lying along the P-loop, while the activation loop stayed in the inactive form. Compound F179, with a carbonyl group in the middle of the molecule, altered the αC helix conformation by interacting with the invariant Lys123. Compounds F176 and F177 bound slightly distant from the hinge region, and their sulfoamide groups formed polar interactions with the protein. The structural features required for the specific binding of inhibitors are discussed.

## Introduction

The 70 kDa ribosomal protein S6 kinase 1, S6K1, is a Ser/Thr protein kinase and a member of the AGC (protein kinases A, G, and C) kinase family. S6K1 plays a key role as a downstream effector in the PIK3/mTOR signaling pathway, which responds to nutrition and growth factor/hormone inputs, and is implicated in cell growth and proliferation [1]. S6K1 was first identified as a protein kinase that phosphorylates ribosomal protein S6 in the 40S ribosomal subunit. The substrates of S6K1 include translation factors such as eIF4B and SKAR, which participate in splicing, and IRS1 in the negative feedback regulation of insulin signaling [2, 3]. S6K1 is involved in a number of functions with various disease-related aberrations, including diabetes, obesity and cancer [3].

S6K1 is activated by phosphorylation on multiple sites. Thr252, in the activation loop of the kinase domain, is phosphorylated by PDK1 [4, 5]. Thr412, in the hydrophobic motif, is phosphorylated by mTORC1 [6, 7]. Ser394, in the turn motif, as well as four Ser residues in the C-terminal regulatory domain, must also be phosphorylated for the full activation of S6K1. However, it has not been confirmed whether the phosphorylation of the activation loop requires the phosphorylation of the hydrophobic motif [8, 9].

S6K1 is one of the two members of the 70 kDa S6 kinase (p70S6K) family in mammals, and shares 84 % sequence identity with the other member, S6K2, in their kinase domains. S6K1 has two isoforms produced by the same gene (*RPS6KB1*) with alternative translational start sites. The longer form, with 525 residues, is termed p85S6K1 (p70S6KαI), while the shorter predominant form, with 502 residues, is termed p70S6K1 (p70S6KαII). The shorter form lacks the N-terminal 23 residues, which contain a nuclear localization sequence [10]. The kinase domains of the two S6K1 isoforms share identical sequences (the S6K1 kinase domain or S6K1KD). We hereafter use the numbering based on the longer 525-residue protein (UniProt P23443).

The development of S6K1 inhibitors has only recently been initiated. The inhibitors include 4-(benzimidazol-2-yl)-1,2,5-oxadiazol-3-ylamine derivatives [11], a piperazinyl-pyrimidine analogue PF-4708671 [12], the 4-phenyl-1*H*-pyrazole derivative AT7867 [13], thiophene urea-templated inhibitors [14], and pyrazolopyrimidines [15, 16]. The structure of S6K1KD complexed with the non-specific inhibitor staurosporine was the first to be reported [17]. Recently, the structures of S6K1 complexed with PF-4708671 were published, with either the S6K1KD protein or the protein with the C-terminal extension including the hydrophobic motif (S6K1HM) and its derivatives [9].

In the present study, we performed an in silico screening of a chemical library followed by a kinase assay, and thereby identified new inhibitors of S6K1. We determined their crystal structures in complexes with S6K1KD.

## Materials and methods

### In silico screening

In the first round of in silico screening, protein–ligand docking, 3D similarity, and 2D substructure searches were employed. First, protein–ligand docking was performed using Glide (Schrödinger, New York, NY) and k-PALLAS [18], to optimize the docking conditions for efficient screening. As the protein datasets, three crystal structures of S6K1KD (PDB: 3A60, 3A61, and 3A62) [17] were used, as well as homology models built with MOE (CCG, Montreal, Canada), based on 23 highly homologous proteins selected by BLAST. For the ligand datasets to validate the screening efficiency, 38 known S6K1 inhibitors (IC_50_ < 10 μM) were collected from the ChEMBL (https://www.ebi.ac.uk/chembl/) and Thomson Reuters Integrity (http://integrity.thomson-pharma.com) databases; in addition, 1,368 decoy compounds were selected from the ZINC drug-like database [19] by using the DUD selection method [20]. Two protein structures were selected as the optimized docking conditions, considering enrichment factors, ROC scores, and X-ray pose reproducibility. Docking simulations were performed using a library composed of 42,792 compounds (the Open Innovation Center for Drug Discovery at the University of Tokyo; http://www.ocdd.u-tokyo.ac.jp). Second, 3D similarity searches, based on molecular shape and pharmacophore locations, were performed with ROCS (OpenEye Scientific Software, Santa Fe, NM). As a result, 11 kinase ligands in crystal structures and 28 known S6K1 inhibitors were selected as queries. Third, a substructure search was performed using Pipeline Pilot (Accelrys, San Diego, CA). The substructure queries were selected from the common substructures of known inhibitors against human S6K1 and homologous proteins. The other queries were manually created, based on kinase-like scaffolds.

In the second round of in silico screening, a 2D similarity search was performed by using the 28 queries with IC_50_ values less than 10 μM, identified in the first kinase mobility shift assay. The compound library used in the first round was also used in the second round.

### Kinase mobility shift assay

The mobility shift assay was performed as reported [21]. Briefly, the reaction buffer consisted of 50 mM Hepes (pH 7.4), containing 10 mM MgCl_2_, 0.01 %(w/v) Brij-35, 1 mM dithiothreitol (DTT), 1 %(v/v) Protease Inhibitor Cocktail Set V [Calbiochem (EMD Millipore), Darmstadt, Germany], 1 %(v/v) Phosphatase Inhibitor Cocktail Set III [Calbiochem (EMD Millipore), Darmstadt, Germany], and 0.6 %(v/v) DMSO. For the kinase assay, the human S6K1 (RPS6KB1) protein, residues 1–421 with the T412E mutation, was purchased from Carna Biosciences (Kobe, Japan). We used the longer protein including the hydrophobic motif, rather than the kinase domain used for the structural study, because the kinase domain alone exhibited low activity [17]. The S6K1 protein (final concentration of 2 nM) in the reaction buffer was first incubated with different concentrations of the compounds (0–10 μM) for 30 min. After this pre-incubation step, a FAM-labeled substrate peptide, FL-Peptide 26 (5-FAM-ARKRERTYSFGHHA-COOH) (PerkinElmer, Waltham, MA), and ATP were added at final concentrations of 1.5 μM and 0.5 mM, respectively. The kinase reactions were performed at room temperature, and stopped by the addition of termination buffer [50 mM Hepes buffer (pH 7.4), containing 140 mM EDTA and 0.01 %(w/v) Brij-35]. The phosphorylated and unphosphorylated peptides thus obtained were separated and quantified with a Lab-Chip EZ Reader II (PerkinElmer, MA). Data were fitted using a sigmoidal dose–response regression algorithm in GraphPad Prism (GraphPad Software, La Jolla, CA).

### Protein expression and purification

The human *RPS6KB1* gene fragment, encoding residues 75–399, was PCR-amplified and cloned into the pDEST 10 vector (Invitrogen). The plasmid thus generated was transformed into DH10 Bac competent cells, and the recombinant bacmid was extracted and transfected into Sf9 insect cells. The Sf9 cells were infected with the baculovirus at a multiplicity of infection of one, and were incubated at 27 °C for 48 h. The Sf9 cells were collected, frozen, and stored at −80 °C until use. The cells were lysed using a sonicator, and the supernatant was applied to a HisTrap column (GE Healthcare). The target protein was eluted and treated with λ protein phosphatase, to remove non-specific phosphorylation, and the N-terminal 6× His tag was removed by TEV digestion. The protein was reloaded on the HisTrap column to remove the undigested protein, and the flow-through fractions were further purified by chromatography on a HiTrap SP column (GE Healthcare) and a HiLoad 16/60 Superdex 75 column (GE Healthcare).

Phosphorylation of the purified S6K1KD protein, using PDK1, was performed as previously described [17]. The His-tagged PDK1 protein was produced using a baculovirus expression system, and was purified prior to addition to S6K1KD.

### Compounds

PF-4708671 was purchased from Tocris Bioscience (Bristol, UK). F108, F109, F176, and F177 were purchased from Enamine (Monmouth Jct., NJ), and F179 was obtained from Pharmeks (Moscow, Russia).

### Crystallization

The protein (15 mg/ml) was incubated with the inhibitors (molar ratio 1:4) overnight before the crystallization setup. Crystals were grown by the sitting drop method at 20 °C, with a reservoir solution of 0.1 M Tris–HCl buffer (pH 8.5) containing sodium formate. The concentrations of sodium formate used for data collections were 2.9–3.3 M for the F108 and F109 complexes, 2.9 M for the F176 and F177 complexes, and 3.7–3.9 M for the PF-4708671 and F179 complexes. Crystals were cryoprotected in well solution containing 15 % (v/v) ethylene glycol, and flash-cooled in liquid nitrogen.

### Data collection, structure determination and refinement

The diffraction data for the F108, F109 and F177 complexes were collected on BL41XU, BL26B2, and BL38B1, respectively, at SPring-8 (Harima, Japan). The data for the F176 and F179 complexes were collected on the MX2 beamline at the Australian Synchrotron (Melbourne, Australia), and those for the PF-4708671 complex were collected on BL1A at the Photon Factory, KEK (Tsukuba, Japan). The data were processed using the *HKL*-2000 program [22] and the CCP4 suite [23]. Molecular replacement was performed with *PHASER* [24], using the coordinates of the protein portion of the phosphorylated S6K1KD·staurosporine complex (PDB: 3A62) [17] as the initial search model. Model building was accomplished with *COOT* [25], and refinement was performed with *PHENIX* [26] using TLS refinement. The topology and parameter files for each inhibitor were generated with the *eLBOW* module of *PHENIX*. The models in the figures were depicted using *PyMOL* (http://www.pymol.org).

### X-ray fluorescence measurement and XAFS

An X-ray fluorescence measurement was performed on a crystal of the S6K1KD·F179 complex, using the MX2 beamline at the Australian Synchrotron (Melbourne, Australia). The fluorescence measurement was performed with an excitation energy of 13 keV. After the zinc was identified, Zn XAFS scans were performed for crystals of each inhibitor complex, mounted for diffraction tests at the SPring-8 beamlines (Harima, Japan).

## Results and discussion

### Inhibitors of S6K1

**Fig. 1:**
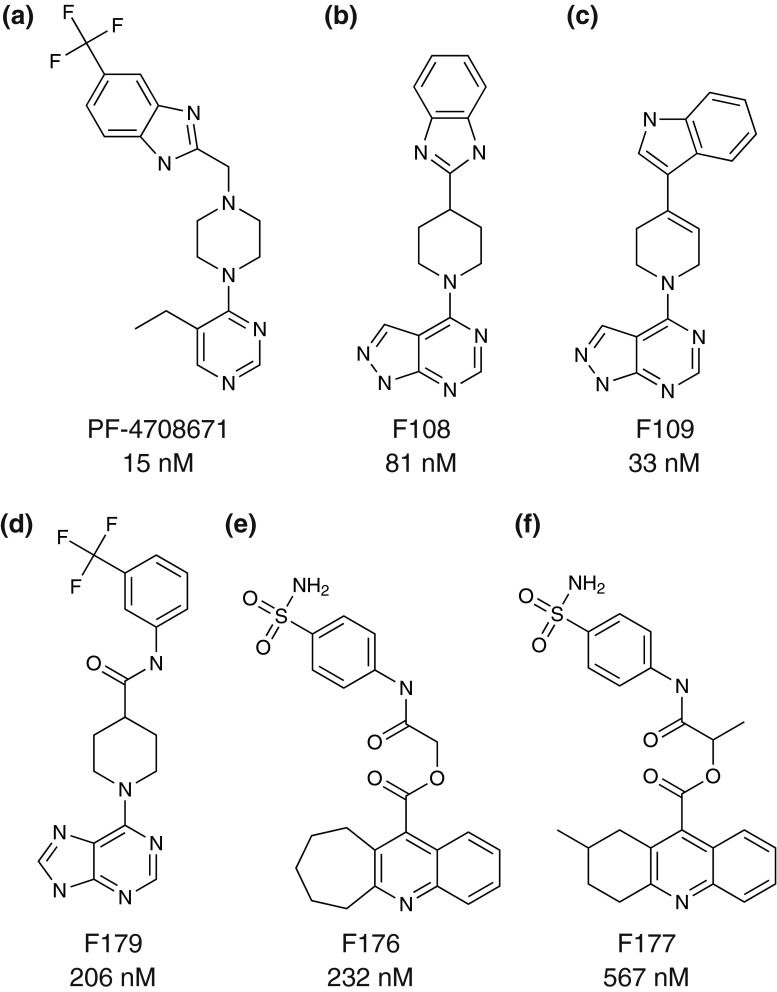
Inhibitors used in this study. IC_50_ values are shown below the names of the inhibitors. Note that the protein composed of residues 1–421 and with the T412E mutation was used for the assay

In the first round of in silico screening, 1,258 candidate compounds were selected. Among them, 595, 436, and 239 compounds were from the docking search, the 3D similarity search, and the 2D substructure search, respectively. The candidate compounds were screened by the kinase mobility shift assay, and four compounds exhibited IC_50_ values less than 0.2 μM. Among them, 4-[4-(1*H*-benzimidazol-2-yl)piperidin-1-yl]-1*H*-pyrazolo[3,4-*d*]pyrimidine (F108) and 4-[4-(1*H*-indol-3-yl)-3,6-dihydropyridin-1(2*H*)-yl]-1*H*-pyrazolo[3,4-*d*]pyrimidine (F109) were selected as S6K1 inhibitors. In the second round, 1,013 compounds were selected by the 2D similarity search, and were screened by the mobility shift assay. Thus, three hit compounds with the lowest IC_50_ values, 2-oxo-2-[(4-sulfamoylphenyl)amino]ethyl 7,8,9,10-tetrahydro-6*H*-cyclohepta[*b*]quinoline-11-carboxylate (F176), 1-oxo-1-[(4-sulfamoylphenyl)amino]propan-2-yl-2-methyl-1,2,3,4-tetrahydroacridine-9-carboxylate (F177), and 1-(9*H*-purin-6-yl)-*N*-[3-(trifluoromethyl)phenyl]piperidine-4-carboxamide (F179), were selected as S6K1 inhibitors. The five inhibitors were used for structural studies. The published S6K1-specific inhibitor, PF-4708671 [12], was also used in this study. Their chemical formulas and IC_50_ values are shown in Fig. 1.

### Crystallization and structure determination

The crystals of S6K1KD cocrystallized with F108, F109, F179, and PF-4708671 exhibited octahedral shapes with the space group *P*4_1_2_1_2, and the unit cell parameters were similar to those of the S6K1KD·staurosporine crystal (PDB: 3A62) [17]. Although the crystallizations occurred under similar conditions, the crystals cocrystallized with F176 and F177 exhibited small plate-like appearances, and the space group was processed as *C*2. All crystals had one protein molecule in the asymmetric unit. The data processing and refinement statistics are summarized in Table 1.

**Table 1:** X-ray data and refinement statistics

	S6K1KD·PF4708671	S6K1KD·F108	S6K1KD·F109
Crystal parameters			
Space group	*P*4_1_2_1_2	*P*4_1_2_1_2	*P*4_1_2_1_2
Cell dimensions			
*a*, *b*, *c* (Å)	70.9, 70.9, 146.9	69.2, 69.2, 143.2	69.1, 69.1, 145.0
* α*, *β*, *γ* (°)	90, 90, 90	90, 90, 90	90, 90, 90
Data collection			
Wavelength (Å)	1.0000	1.0000	1.0000
Resolution (Å)	36–2.00 (2.03–2.00)	41–2.10 (2.14–2.10)	35–2.03 (2.07–2.03)
No. of unique reflections	26,174	20,923	22,671
Mean redundancy	9.5 (9.6)	12.1 (12.4)	13.7 (14.6)
Overall completeness (%)	99.3 (100.0)	98.9 (98.2)	96.5 (98.9)
*R*_sym_ (%)	12.0 (>100.0)	8.1 (>100.0)	4.7 (>100.0)
Mean *I*/*σ*	13.3 (2.0)	30.0 (2.0)	43.1 (2.3)
Refinement residuals			
*R*_free_ (%)	23.1	23.3	26.5
*R*_work_ (%)	17.5	17.5	19.5
Model quality			
RMSD bond lengths (Å)	0.007	0.008	0.008
RMSD bond angles (°)	1.011	1.054	1.058
Mean protein B-factor (Å^2^)	50.8	49.9	64.4
Mean water B-factor (Å^2^)	55.0	49.6	55.7
Mean ligand B-factor (Å^2^)	39.7	37.8	50.2
Model contents			
Protomers in ASU	1	1	1
No. of protein atoms	2,286	2,220	2,221
No. of water molecules	183	110	59
No. of ligand atoms	28	24	24
PDB accession code	3WE4	3WF5	3WF6

	S6K1KD·F179	S6K1KD·F176	S6K1KD·F177
Crystal parameters			
Space group	*P*4_1_2_1_2	*C*2	*C*2
Cell dimensions			
* a*, *b*, *c* (Å)	69.4, 69.4, 145.7	121.7, 62.5, 80.0	121.5, 62.3, 79.9
* α*, *β*, *γ* (°)	90, 90, 90	90, 128.0, 90	90, 128.2, 90
Data collection			
Wavelength (Å)	0.9537	0.9537	1.0000
Resolution (Å)	49–1.85 (1.88–1.85)	40–1.97 (2.00–1.97)	35–2.04 (2.08–2.04)
No. of unique reflections	31,350	33,311	29,807
Mean redundancy	9.6 (9.7)	3.8 (3.3)	4.0 (3.8)
Overall completeness (%)	100.0 (100.0)	98.8 (77.0)	98.6 (97.8)
*R*_sym_ (%)	8.1 (>100.0)	13.9 (66.2)	14.1 (70.8)
Mean *I*/*σ*	26.3 (1.9)	8.9 (2.1)	9.6 (2.3)
Refinement residuals			
*R*_free_ (%)	22.4	24.1	26.5
*R*_work_ (%)	17.3	20.1	21.4
Model quality			
RMSD bond lengths (Å)	0.007	0.007	0.008
RMSD bond angles (°)	0.967	0.991	1.039
Mean protein B-factor (Å^2^)	42.7	29.9	31.6
Mean water B-factor (Å^2^)	49.5	41.8	39.1
Mean ligand B-factor (Å^2^)	43.2	31.4	30.4
Model contents			
Protomers in ASU	1	1	1
No. of protein atoms	2,309	2,295	2,295
No. of water molecules	226	321	289
No. of ligand atoms	28	32	33
PDB accession code	3WF7	3WF8	3WF9

### Overall structures

**Fig. 2:**
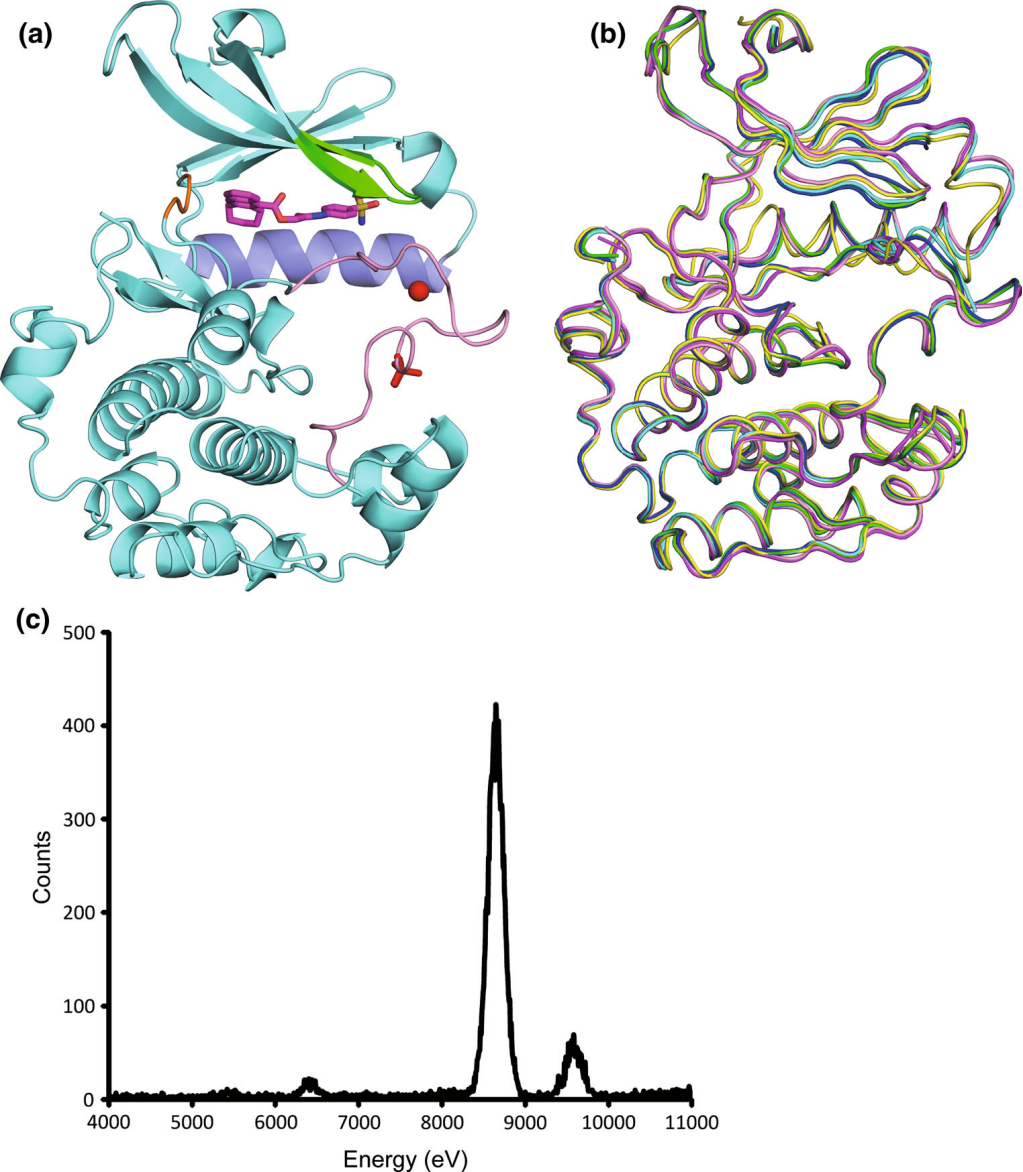
Structure of S6K1KD·F176. **a** The protein is shown as a *ribbon model in cyan*, with the P-loop and the strands β1 and β2 in *green*, helix αC in *pale blue*, the hinge region in *orange*, and the activation loop in *pink*. The phosphorylated Thr252 is depicted by *red sticks*, and the zinc ion is a *red sphere*. The bound inhibitor (F176) is shown in *magenta*. **b** Superimposition of the Cα traces of the six protein structures. The structures are colored as follows: *cyan*, S6K1KD·PF-4708671; *green*, S6K1KD·F108; *blue*, S6K1KD·F109; *magenta*, S6K1KD·F179; *yellow*, S6K1KD·F176; *pink*, S6K1KD·F177. **c** X-ray fluorescence measurement from an S6K1KD·F179 crystal

The crystal structures of S6K1KD with the inhibitors exhibited the typical bilobal structures of protein kinases, and the inhibitors occupied the ATP-binding pocket between the small N-terminal lobe and the large C-terminal lobe (e.g. F176 in Fig. 2a). Although the F176 and F177 complexes were crystallized in a different packing mode from that of the others, they shared similar overall conformations (Fig. 2b). The F179 complex has a different αC conformation as compared with the others, as described below in detail. Correspondingly, the Cα root mean square deviation (rmsd) values of the structures of the F108, F109, F176, F177, and F179 complexes with that of the PF-4708671 complex are 0.43, 0.41, 0.69, 0.70, and 1.05 Å, respectively, indicating a slightly larger difference for the F179 complex structure. The F108 and F109 complex structures were almost identical to each other (Cα rmsd value, 0.27 Å), and so were the F176 and F177 complex structures (Cα rmsd value, 0.17 Å).

In these S6K1KD structures, a strong electron density peak was observed in the Fourier difference maps and anomalous difference Fourier maps. This peak was assigned to zinc for the PF-4708671 complex [9]. To confirm this assignment, we performed an X-ray fluorescence measurement on the S6K1KD complex crystal (Fig. 2c). Two significant peaks were detected at 8.65 and at 9.58 keV, which were quite consistent with the theoretical values of zinc Kα (KαI 8.64 keV and KαII 8.62 keV) and Kβ (9.57 keV), respectively. Therefore, it was unambiguously concluded that the element was zinc. Subsequent XAFS analyses revealed that zinc existed in all of the S6K1KD complex structures in this study. The zinc-binding site is composed of the C–H–H–C residues, and is located in the middle of the activation loop [9] (Fig. 2a).

### Inhibitor binding

**Fig. 3:**
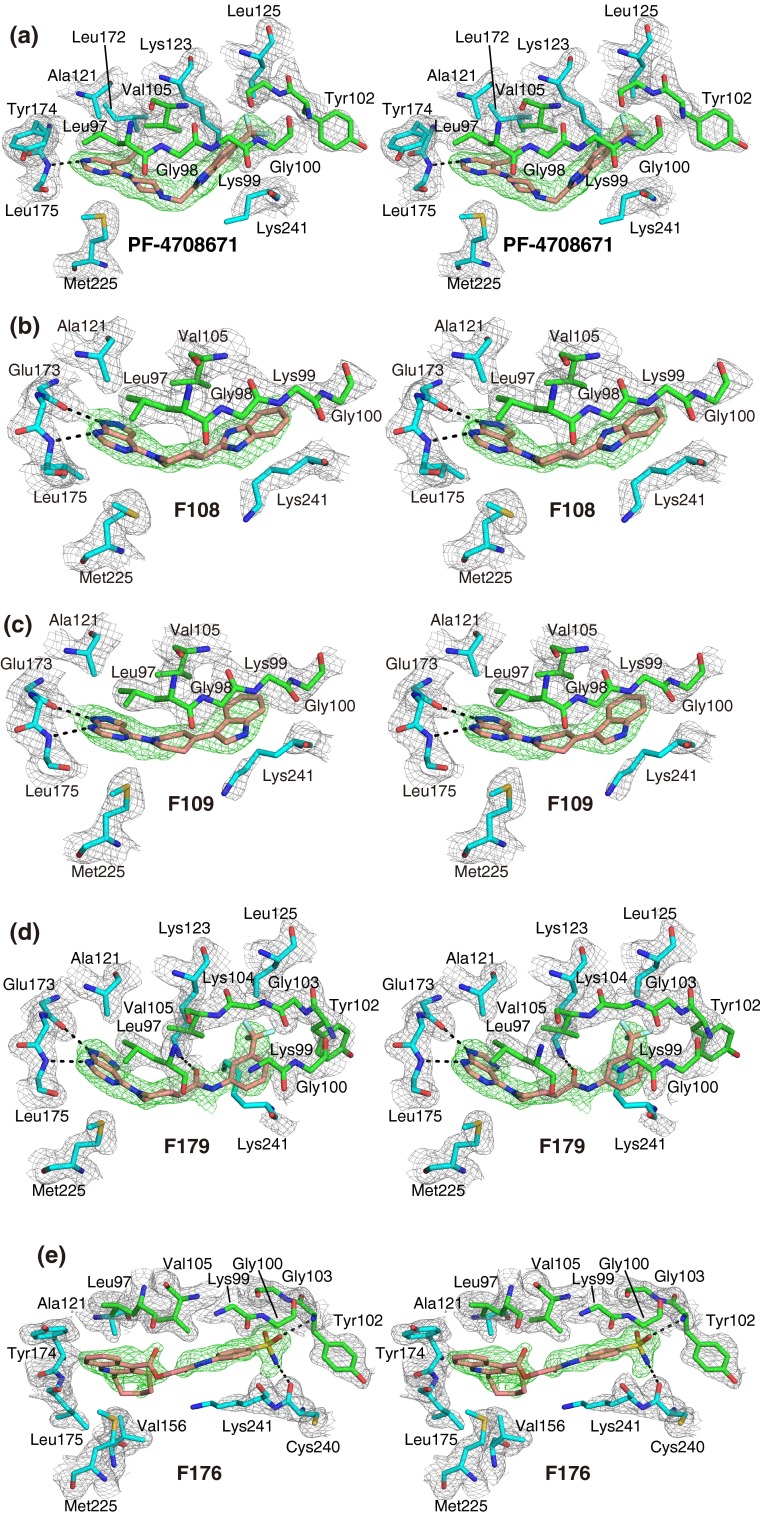

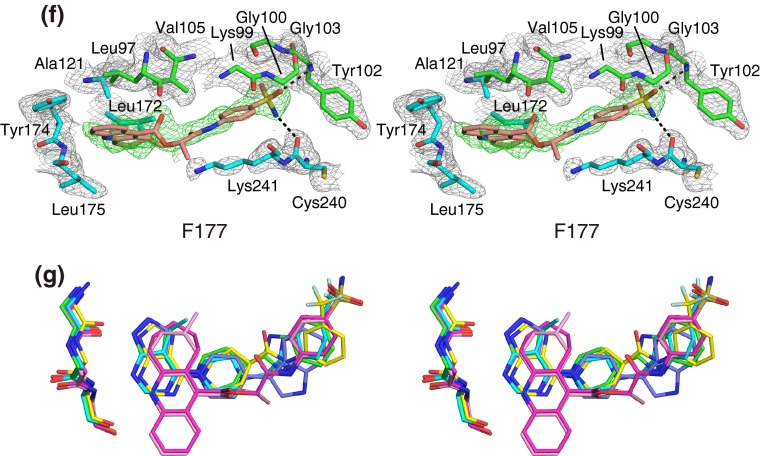
Inhibitor binding (stereoviews). **a** S6K1KD·PF-4708671 in this study, **b** S6K1KD·F108, **c** S6K1KD·F109, **d** S6K1KD·F179, **e** S6K1KD·F176, **f** S6K1KD·F177. In (**a**)–(**f**), the protein residues are *colored cyan*, and the residues in the P-loop and the strands β1 and β2 are *green*. Inhibitors are shown in *salmon*. The simulated-annealing composite omit maps (2*mF*o-*DF*c) are contoured at 1.2σ and depicted around the inhibitors (*green mesh*) and protein residues (*gray mesh*). These interactions were analyzed with LIGPLOT [28] and by manual inspection. **g** Superimposed bound inhibitors (stereoview). The color scheme is the same as in Fig. 2b. The backbones of the hinge region are also shown, with the same color scheme

The inhibitor binding modes of S6K1KD, shown in Fig. 3a–f, share some common features. One or two aromatic rings, shown at the bottom of each panel in Fig. 1, face the hinge region of the kinase domain, and are surrounded by hydrophobic residues, such as Leu97 and Ala121 at the top and Met225 at the bottom. The inhibitor molecules form extensive hydrophobic and van der Waals interactions between strands β1 and β2, and interact with their residues, such as Leu97 and Val105. These β-strands are just before and after the P-loop in the sequence. The aromatic ring(s) at the other end of the inhibitor molecules reach or approach the P-loop side at the top and Lys241 at the bottom. Thus, the inhibitor molecules extend from the hinge region, lie alongside the β1–P-loop–β2 moiety, and bend in the middle. The inhibitors are not bound in the ATP phosphate-binding site, which is occupied by the side chain of Lys241. Additional details for each inhibitor are described, as follows.

The structure of S6K1KD·PF-4708671 (Fig. 3a) is almost identical to the recently published structure of S6K1KD·PF-4708671 (PDB: 4L3J) [9], with a Cα rmsd value of 0.3 Å. The inhibitor interacts with the hinge region, and its pyrimidyl nitrogen atom forms a hydrogen bond with the amide nitrogen atom of Leu175 (Fig. 3a). The ethyl group is located in the hydrophobic environment formed by Val105, Ala121, and Leu172. The trifluoromethyl group contacts the side chains of Leu125 and Lys123 and the main chain of Gly103.

The pyrazolopyridine ring of F108 forms two hydrogen bonds with the hinge (Fig. 3b); a pyrimidyl nitrogen atom hydrogen bonds with the nitrogen atom of Leu175, while a pyrazole nitrogen atom hydrogen bonds with the carbonyl oxygen atom of Glu173. The piperidine–benzimidazole moiety of F108 interacts with Leu97 and Val105 from strands β1 and β2, respectively. F109 interacts with the protein in basically the same manner as F108 (Fig. 3c). The purine ring of F179 hydrogen bonds with the nitrogen atom of Leu175 and the carbonyl oxygen atom of Glu173 (Fig. 3d). The trifluoromethyl group contacts the side chains of Leu125 and Lys241 and the main chains of Gly103 and Lys104. The carbonyl oxygen atom, in the middle of F179, hydrogen bonds with Nε of Lys123.

F176 also binds to the ATP binding site. However, it forms only weak interactions with the hinge, and the distance between the nitrogen atom in the cycloheptaquinoline ring and the nitrogen atom of Leu175 is 3.5 Å (Fig. 3e). The cycloheptyl ring forms extra hydrophobic interactions with Val156. The sulfoamide group at the other end of the molecule forms hydrophilic interactions with the P-loop and the activation loop, by hydrogen bonding with the nitrogen atom of Tyr102 and the carbonyl oxygen atom of Cys 240. F177 binds to the protein in a similar manner to that of F176 (Fig. 3f). The methyl group in the middle of F177 protrudes toward the solvent region. The methyltetrahydroacridine ring of F177 forms hydrophobic interactions with Leu172, instead of the interaction between the cycloheptyl ring of F176 and Val156. A superimposition of the bound inhibitors, shown in Fig. 3g, reveals the positional shifts of F176 and F177 in comparison with the other inhibitors.

As compared with PF-4708671, the five inhibitors found in this study have lower potencies, in terms of the IC_50_ values ranging from two- to more than 40-fold (Fig. 1). PF-4708671 has an ethyl group on the pyrimidine ring, which is accommodated in the hydrophobic pocket formed by Val105, Ala121, and Leu172, next to the hinge-binding site, while F108 and F109 have fewer hydrophobic interactions in these regions. F179 has a purine as the hinge-binding group, instead of the pyrazolopyrimidine in F108 and F109. Replacing the pyrazolopyrimidine moiety with purine resulted in a two-fold loss of activity [15], which might be relevant to the weaker binding of F179 as compared to those of F108 and F109. F176 and F177 form weaker interactions with the hinge region than the other inhibitors, which may be the reason for their relatively less robust binding activities.

### Conformational changes in the S6K1KD·F179 complex

**Fig. 4:**
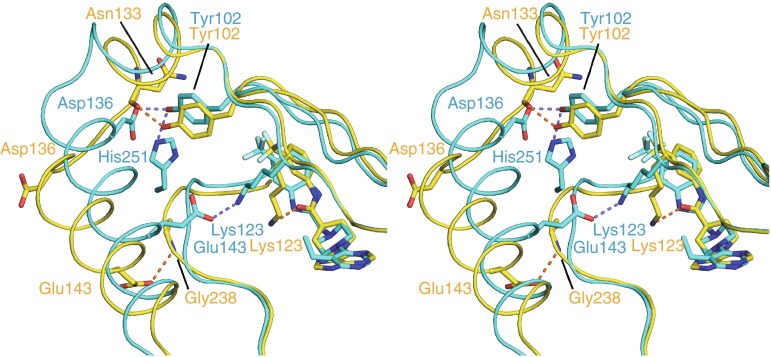
Conformational changes in the S6K1KD·F179 complex (stereoview). Superimposition of S6K1KD·F179 (*yellow*) and S6K1KD·PF-4708671 (*cyan*)

In the S6K1KD·F179 complex structure, the carbonyl group in the middle of the inhibitor interacts with the Nε of Lys123 (Fig. 3d). This is the invariant lysine residue that interacts with Glu143 in helix αC, in the active form of the kinases [27]. In the other S6K1KD complexes in this study, Lys123 interacts with Glu143. The Nε atom of Lys123 in the S6K1KD·F179 structure moves by 3.0 Å towards the carbonyl group, as compared with the S6K1KD·PF-4708671 structure. Along with the rotation, helix αC moves down by about 3 Å, and thus Glu143 can hydrogen bond with the nitrogen atom of Gly238 (Fig. 4). Ala134 and Lys135 form the N-terminal cap of helix αC in the complex structures with PF-4708671, F176, and F177, where the side chain of Asp136 interacts with the hydroxyl group of Tyr102 in the P-loop. In the S6K1KD·F179 complex structure, however, Ala134 and Lys135 do not adopt the helical conformation and Asp136 moves away from Tyr102. Instead, the carbonyl oxygen atom of Asn133 interacts with Tyr102 (Fig. 4).

### Inter- and intra-molecular interactions in the S6K1KD·F176/F177 complexes

**Fig. 5:**
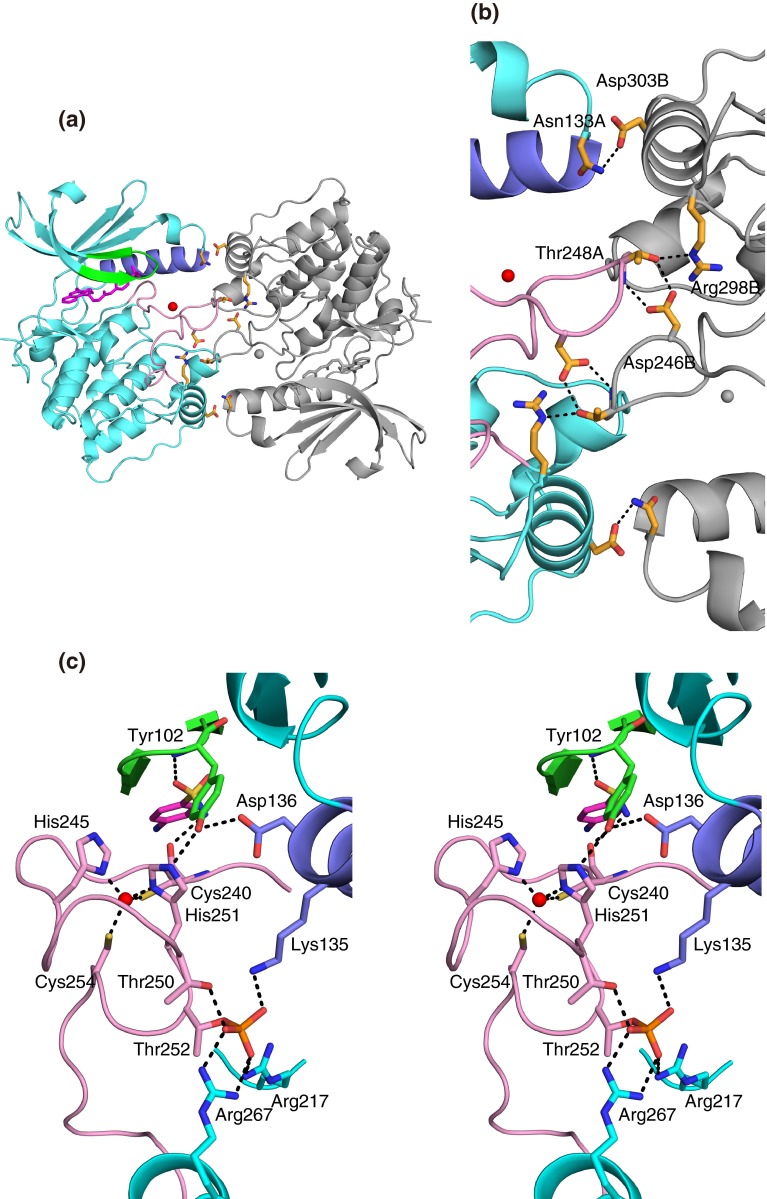
Interactions of the S6K1KD·F176 complex. **a** Crystallographic two-fold related S6K1KD·F176 complexes. One molecule is *colored* in the same manner as in Fig. 2a. The other complex is colored *gray*. Interacting residues are shown as *orange sticks*. **b** Close-up view of the interface. **c** Interactions of the inhibitor, Tyr102, zinc ion, and phosphorylated Thr252 (stereoview). Interacting residues are shown as *sticks*, *colored* in the same manner as in (**a**)

In the S6K1KD·F176 crystal, the complex molecule contacts the symmetry-related molecule, with their activation loops facing each other, across one of the dyad axes of the *C*2 space group (Fig. 5a). At the interface (Fig. 5b), Thr248 in the activation loop interacts with Asp246 in the activation loop and Arg298 in αG of the other molecule. In addition, Asn133 in the loop between αB and αC interacts with Asp303 in αG of the other molecule.

Inside the F176 complex (Fig. 5c), Tyr102 in the P-loop hydrogen bonds with the side chains of Asp136 in helix αC and His251 in the activation loop, while His251 and Cys240 coordinate the zinc ion, together with His245 and Cys254 in the activation loop. This scheme was also observed in the S6K1HM·PF-4708671 and S6K1KD·PF-4708671 complexes [9]. In the F176 complex, the inhibitor additionally forms hydrogen bonds with the main-chain atoms of Tyr102 and Cys240, as described above (Figs. 3e, 5c). The phosphate group of the phosphorylated Thr252 interacts with Thr250, Arg217, and Arg267, which are also observed in the complex structures in the present study. In the S6K1KD·F176 structure, Lys135 in αC, which is in the region buried by the symmetry-related molecule, also participates in the interactions with the phosphorylated Thr252 (Fig. 5c).

These inter- and intra-molecular interactions seem to stabilize the conformations of helix αC and the activation loop of the S6K1KD·F176 complex, as indicated by the low B-factor values of 29.9, 31.3, and 31.7 Å^2^ for the overall structure, helix αC (134–150) and the solvent-exposed moiety of the activation loop (240–256) of the S6K1KD·F176 structure, respectively, in contrast to those of 50.8, 66.9 and 77.6 Å^2^ for the counterparts of the S6K1KD·PF-4708671 structure (residues 247–249 were not modeled, due to disorder). These interactions and the lower B-factors are also observed in the S6K1KD·F177 structure.

### Inactive form of the activation loop

The protein structures presented here may be considered as partially active. The DFG motif assumes the so-called DFG-in conformation (not shown), a feature of the active form. The salt bridge between Lys123 and Glu143, a hallmark of the active form of protein kinases [27], was observed in all of the structures except for the F179 complex, in which Lys123 interacts with the inhibitor molecule. However, similar to other S6K1KD and S6K1HM structures [9, 17], in all of the structures in this study, the manner in which the activation loop winds through the substrate-binding site would sterically hinder substrate binding. Further studies are necessary to reveal the conformation of the activation loop in the substrate-bound complex.
